# Telenursing on primary family caregivers and children with disabilities: a scoping review

**DOI:** 10.3389/fped.2024.1374442

**Published:** 2025-01-31

**Authors:** Kaori Nishigaki, Noyuri Yamaji, Naho Adachi, Tomoko Kamei, Kyoko Kobayashi, Shota Kakazu, Yuki Yonekura

**Affiliations:** ^1^ Department of Child Health Nursing, Graduate School of Nursing Sciences, St. Luke’s International University, Tokyo, Japan; ^2^ Global Health Nursing, Graduate School of Nursing Science, St. Luke's International University, Tokyo, Japan; ^3^ Family Nursing, Graduate School of Medicine, The University of Tokyo, Tokyo, Japan; ^4^ Department of Child Health Nursing, Graduate School of Nursing Sciences, Aichi Prefectural University, Aichi, Japan; ^5^ Department of Gerontological Nursing, Graduate School of Nursing Sciences, St. Luke’s International University, Tokyo, Japan; ^6^ Department of Nursing Informatics, Graduate School of Nursing Sciences, St. Luke’s International University, Tokyo, Japan

**Keywords:** telenursing, children with disabilities, COVID-19 pandemic, scoping review, home care

## Abstract

**Introduction:**

Despite the need for continued support for children with disabilities and their families, COVID-19 has made the support difficult. Telenursing can monitor daily life and support from a remote location, continuously and at a low cost. However, there are few practical reports on its use in children with disabilities.

**Objective:**

This scoping review aims to summarize the published literature on telenursing for children with disabilities in home care settings.

**Methods:**

We included studies involving children (0–18 years of age) with disabilities and their families and specified telecommunication assessment, monitoring, and intervention such as tele-education, teleconsultation and telementoring involving nurses via information communication technologies. We included studies that used any design written in English or Japanese. A comprehensive search was conducted on March 14, 2021 using six databases: MEDLINE via PubMed, Cumulative Index to Nursing & Allied Health Literature, Cochrane Central Register of Controlled Trials, Embase, PsycINFO, and Ichushi Web. Two or more reviewers individually screened eligible studies according to the Preferred Reporting Items for Systematic Reviews and Meta-Analyses flow diagram. Data on the characteristics of the included studies, telecommunications technology interventions, and children with disabilities were mapped in tables.

**Results:**

Eleven studies, published between 2003 and 2020, met the inclusion criteria. Only one study used the term “telenursing,” while others used “telehealth” and other terms, which involved multidisciplinary support such as physicians and social workers in addition to nurses. Although there were various types of telecommunications technology interventions, they were categorized as consultation, healthcare provision, monitoring, and education. Most studies have examined telecommunication technology interventions by healthcare professionals, including nurses. Five studies focused on children with medical complexities, and two focused on children with neurodevelopmental disabilities, including developmental delays. There is insufficient information on telecommunications technology interventions, especially assessment tools, trigger points, and the status of the target population.

**Conclusion:**

This scoping review aimed to map the published literature on telenursing for children with disabilities and their families in home care settings. Available evidence indicates a lack of research focusing on the implementation of telecommunications for children with disabilities and their families. Further research is required to assess the effects of telecommunications technology interventions. Additionally, they should provide information for implementing telecommunication technology safety.

**Scoping Review Registration:**

Figshare (https://doi.org/10.6084/m9.figshare.21747047.v1).

## 1 Background

Children with disabilities and their families must comprehend the needs that arise during each developmental stage and practice self-care to improve the quality of life (QoL). Nonetheless, the COVID-19 pandemic has presented challenges for families of children with disabilities in obtaining the ongoing support required through direct care (1, 2).

Families of children with neurodevelopmental disabilities, as categorized in the Diagnostic and Statistical Manual of Mental Disorders, Fifth Edition (DSM-5) (3), require specific nurturing skills, and many reports show that they experience a unique sense of burden. People with disabilities are at risk of secondary conditions such as pain, spasticity, and depression, but there are several conditions that can be prevented and managed (4). Therefore, it is essential to reduce the incidence of preventable impairments and conditions (5). However, if a child's supportive environment, such as family and formal services, is inadequate, there is a heightened risk of developing secondary conditions after puberty. Secondary conditions can make it exceedingly difficult for children to reconstruct their lives and adapt to society. Furthermore, the prevalence of developmental disabilities in children is as high as 6.5% in regular elementary and junior high school classes and approximately 10% of children have developmental biases (6). In addition, the onset of developmental disabilities has been linked to epigenetics and is projected to increase in the future. These facts emphasize the pressing need to provide support to children with developmental disabilities who do not have intellectual disabilities.

Asakura estimated that approximately 47,000 children in Japan have severe motor and intellectual disabilities (SMID), with an incidence rate of 0.04%. Of these, approximately 35,000 (69%) received home treatment (7). The number of children enrolled in elementary and junior high schools requiring ongoing medical care after long-term hospitalization in the NICU or other facilities (hereafter referred to as “children requiring medical care”) increased significantly from 5,901 in FY2006 to 8,750 in FY2014. These children are often included in SMID counts. Thus, the number of children with SMID is expected to continue to rise in the future owing to advancements in neonatal and pediatric medicine. Researchers have reported that families of children requiring ongoing medical care face significant physical and mental burdens caused by the constraints such care places on their social participation. Additionally, the physical burden of home treatment on children with disabilities is significant, as are the mental and physical burdens on their families, who must adapt to provide such care (8–10). To cope with this change, it is important to enhance the environment for children and their families through social resources and provide support that aligns with the child's developmental stages.

Scholars have observed that children with disabilities have highly individualized physical and mental conditions, and the difficulty in accessing care from personnel outside the family places a burden on primary caregivers in the family. This can alter how the family functions and diminish the family's overall QoL (10, 11), including that of siblings (12–14). Offering tools that can properly identify individual needs and provide continuous support can enhance family members' daily lives and prevent them from becoming overburdened. Telehealth is defined as the use of electronic information and communications technologies to provide and support health care when distance separates participants (15). And telehealth uses technologies include videoconferencing, the internet, store-and-forward imaging, streaming media, and terrestrial and wireless communications (16). From this perspective, telehealth began to spread with the spread of the Internet in the 1990s, mainly in Europe and the United States (17). The effectiveness of telehealth in managing chronic diseases, preventing exacerbations, and controlling medical costs was verified (18). Today, telenursing is provided by connecting various medical centers (17, 18). Telenursing provides continuous monitoring of physical state and daily life, which is challenging to accomplish using conventional medical interventions that operate within a fixed timeframe, such as home nursing and medical treatment (17, 19). As for telenursing, research efforts are underway to manage people with COPD (20) and to verify its effectiveness in managing patients with chronic diseases and cancer (21, 22). However, in the case of telenursing for children, although there have been reports of providing post-discharge support by telephone in the NICU (23) and monitoring the pain in pediatric rheumatology patients (24), there have been few continuous practical reports on its use in children. Also, particularly those with disabilities and its effectiveness has not yet been adequately examined.

## 2 Review question

What is the scope of the published literature on telenursing for disabled children in home care settings? This scoping review aims to answer the following research questions:

• What are the characteristics of telenursing for children with disabilities and their families?
• What are the outcome measures and endpoints of telenursing for children with disabilities and their families?
• What telenursing protocols are recommended for children with disabilities and their families in Japan?

## 3 Methods

This review was conducted in accordance with Joanna Briggs Institute's (JBI) methodology for scoping reviews (25). The search and screening results were reported according to the Preferred Reporting Items for Systematic Reviews and Meta-Analyses extension for scoping reviews (PRISMA-ScR) (26). The scoping review protocol is registered in figshare (https://doi.org/10.6084/m9.figshare.21747047.v1).

### 3.1 Eligibility criteria

We followed the population-conceptual-context framework recommended by the Joanna Briggs Institute (25).

#### 3.1.1 Participants

We included children with disabilities aged 0–18 years and their families. Children with neurodevelopmental disorders were defined as those who met the diagnostic criteria for intellectual disabilities specified in the DSM-5 (3) and were aware of their developmental biases. Children reliant on medical devices were defined as those with special health needs characterized by clinical weakness, demand for healthcare beyond one offered to children of the same age, and frequent need for technological devices (27). Children in this group required continuous support from medical devices, such as ventilators or tracheostomy tubes, for tube feeding and suctioning. Family was defined as the parents or siblings living with a child with disabilities. Disability was defined as the presence of neurodevelopmental disorders or special healthcare needs.

#### 3.1.2 Concept

We included studies that focused on telenursing including telecommunication, assessment, monitoring, and giving intervention such as tele-education, teleconsultation and telementoring involving nurses via information communication technologies (an intervention) for children under 18 years of age and their families to improve the management of their disabilities and clinical outcomes. We did not have any restrictions on the intervention (e.g., length, frequency, and institution). We followed the definition of telenursing by the International Council of Nurses (ICN) (19), Telenursing guidelines (28) and American Academy of Ambulatory Care Nursing (29). We defined telenursing as “the use of information communication or telecommunications technology in nursing, including the use of electromagnetic channels to transmit voice, data, and video communication signals for the purpose of enhancing patient care.” We included interventions that involved nurses, although telenursing was not used in the primary studies. We excluded telephonic support without monitoring and/or the use of information and communication technologies. In this study, we determined that if a nurse uses some ICT equipment to provide care, even if the expression in the paper is not telenursing but another expression such as telehealth, the nurse falls under the above definition of ICT.

#### 3.1.3 Types of sources

We included studies with research designs published from 2000 to the present (2022; preprints were not considered). Quantitative, qualitative, and mixed-method studies written in English were included. Unpublished or ongoing trials were excluded from the analysis.

### 3.2 Search strategy

The search strategy included keywords and index terms based on population and concepts such as disability and telenursing, and it was adapted for each information source. A comprehensive search was conducted using six databases: MEDLINE via PubMed, Cumulative Index to Nursing & Allied Health Literature (CINAHL), Cochrane Central Register of Controlled Trials (CENTRAL), Embase, PsycINFO, and Ichushi Web. We restricted the studies to those written in Japanese or English. Appendix 1 provides the search strategies for each database. We checked the reference lists of relevant articles and manually searched for articles that met our eligibility criteria.

### 3.3 Study selection

All identified records were uploaded to EndNote v.X9 (Clarivate Analytics, PA, USA). We screened eligible studies following the Preferred Reporting Items for Systematic Reviews and Meta-Analyses (PRISMA) flow diagram and presented the results of the screening (30). All citations were then transferred to Rayyan software (31), which helped with the systematic literature reviews. Duplicate records were removed using the Rayyan software. Seven reviewers (K.N., N.Y., S.K., A.K., K.M., S.E., and N.A.) independently screened titles and abstracts according to the inclusion criteria. The full texts of all the retrieved eligible references were uploaded to Rayyan for the second phase of this review. Three reviewers (K.N., N.Y., and S.K.) screened the full text of selected and retrieved articles. Disagreements between reviewers at any stage of the selection process were resolved through discussion or by a third reviewer.

### 3.4 Data extraction

Data from the included studies were extracted and recorded in a Microsoft Excel (Redmond, Washington, USA) form that was developed for this review. We extracted the following data: characteristics of the included studies (author, publication date, source, setting/location, methodology, outcome, and key findings), characteristics of telenursing (types of telenursing, duration, nurses’ description, and tools for assessment), and characteristics of the population (age, description of the disability, comorbidities, type of school or nursery, use of social services, and family situations). We added the following data: purpose of the study, outcome measurement, provider, control group, procedure, content of monitoring telehealth, trigger points, and population. Disagreements were resolved through discussion or consultation with a third reviewer.

### 3.5 Data analysis and presentation

We present the extracted data in a tabular format in a manner that aligns with the objective of the scoping review. We have presented three tables, including the characteristics of the included studies, telenursing, and children with disabilities. These results were accompanied by a narrative summary describing the actual situation and effect of telenursing on children with disabilities.

### 3.6 Changes from protocol

We modified some of the methods used in the protocol. First, we excluded the phrase “in Japan” from our review. For example, we changed the research question 1 as “What are the characteristics of telenursing for children with disabilities and their families?” from “What are the characteristics of telenursing for children with disabilities and their families in Japan?” Although the former goal of this project was to develop a telenursing system for children with disabilities and their families in Japan, this scoping review focused on a global setting. Because, we believed it was more important to clarify the global situation. It maps existing evidence worldwide. Second, we included studies that researched telehealth and telemedicine if they included nurses in the interventions, because we found a limited number of studies on telenursing. Third, we added additional items and extracted data. The details are provided in the Data Extraction section. Fourth, we modified our analysis method. In the protocol, we performed text mining and qualitative content analysis of the data obtained. However, we found limited evidence supporting this analysis. Thus, we summarized the extracted data in the Table and described it narratively.

## 4 Results

### 4.1 Selection of sources of evidence

**Figure 1:**
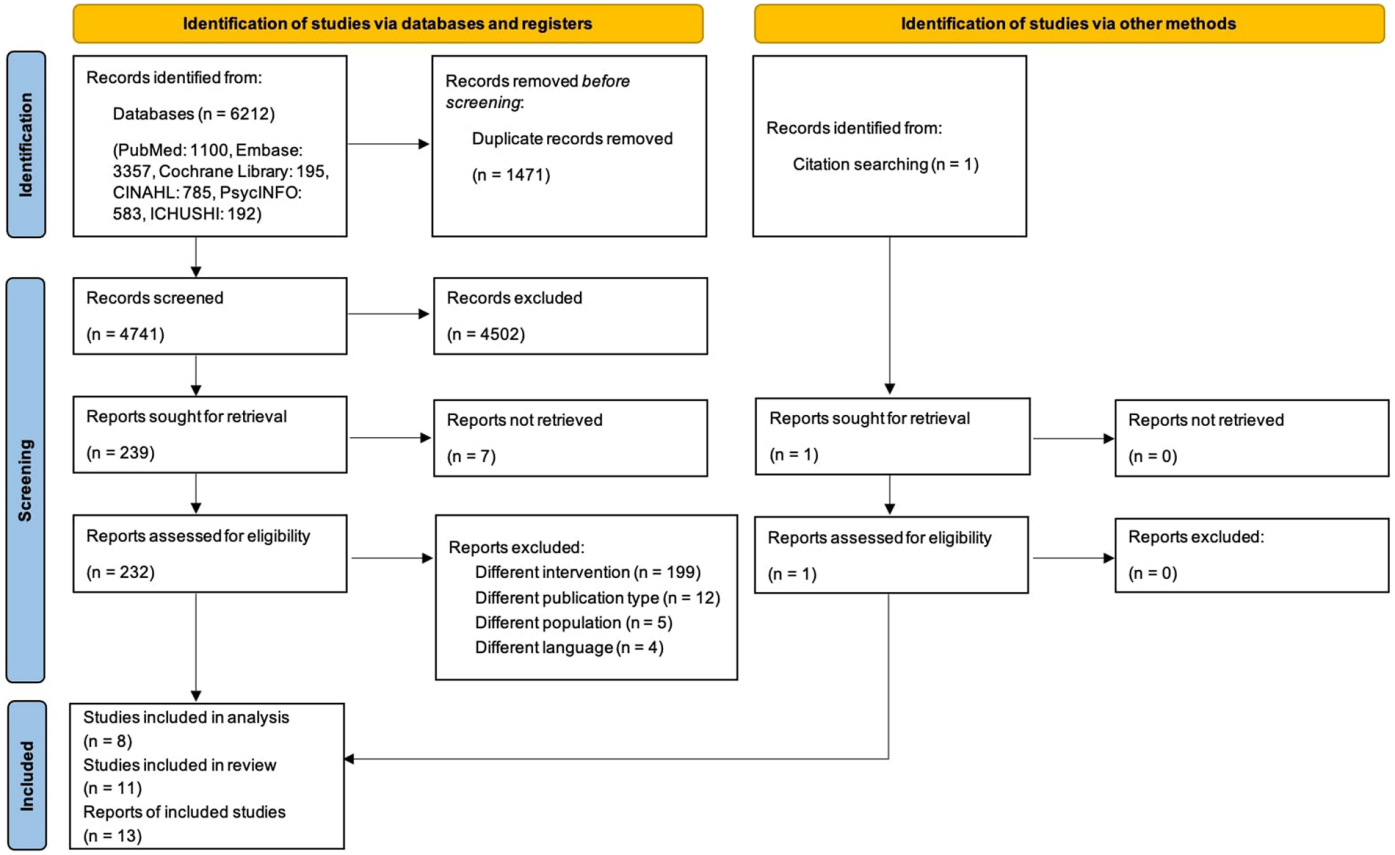
The flow diagram of the search and study selection process.

The screening process is shown in Figure 1. Electronic database searches generated 6,212 references. Duplicates (1,471) were removed, and 4,741 references were assessed for eligibility by title and abstract screening. After excluding seven references that were not retrieved, 232 references were assessed for eligibility by full-text screening. We excluded 219 references due to different interventions, publication types, populations, and languages. One reference was identified by manual search (32). We included a total of 13 references that met our inclusion criteria (32–44). Since Cady et al. and Cady et al. were the same project (36, 37), and Dick et al. and Young et al. obtained the same results (32, 44), the total number of studies was 11. We excluded three papers from the mapping process because they did not report specific telenursing interventions (33–35).

### 4.2 Characteristics of the included studies

Table 1 summarizes the characteristics of the included studies. The selected articles were published between 2003 and 2020. Three articles provided an overview of telehealth and were categorized as reports (33–35). Although all the included studies utilized telecommunication technologies to improve the care of children and their families, only one referred to the practice of “telenursing” (34). Other interventions were administered. Specifically, three studies referred to the practice as “telemedicine” (35, 39, 40), two used “telehealth” (33, 38), and other used various terms, including, “tele-home-care” (32, 42) and “telephone nursing line” (41). Two studies did not describe the specific terminology used in telecommunications technology (37, 43). Three studies aimed to describe the interventions (38, 39, 42), four investigated the feasibility of the interventions (32, 37, 40, 41), and one evaluated the efficacy of the intervention using a control group (43). Five studies were conducted in the United States of America (USA) (33–36, 40), two in Canada (32, 41), one in the United Kingdom (39), and one in Japan (42); two did not report the countries studied (38, 43). The target population comprised children with disabilities (11 studies) (33–35, 37, 39, 41, 42) and their families (*n* = 4) (32, 38, 40, 43). Although Barry et al. focused on children with disabilities, there was no specific description of the types of disabilities (33). Bennett et al. studied obesity among the youth with intellectual and developmental disabilities (34). Most studies were not experimental; thus, there was no control group. Toly et al. reported that, although the intervention group was provided with a resourcefulness training intervention online, both the intervention and control groups used telephones (43). Harper compared satisfaction in parents/caregivers and professionals with experience with telemedicine encounters and those without experience, as well as those with prior clinical experience with the collaboration between hospital treatment systems (40). Although some studies did not specify the study design, they were heterogeneous, comprising six observational studies (37–42) and two experimental studies (randomized controlled trials and before-and-after studies) (32, 43).

**Table 1:** Characteristics of included studies.

Author, year	Purpose of study	Setting/location	Study population	Study design	Outcome	Outcome measurement	Key findings
Barry, 2018 (33)	Not specified.	USA	Children with disabilities	Not specified.	NA	NA	NA
Bennett, 2017 (34)	To highlight studies on treatment of obesity among youth with intellectual and developmental disabilities.	USA	Obesity among youth with intellectual and developmental disabilities.	Not specified.	NA	NA	• All interventions were delivered in-person, and further development of promising approaches and delivery via telenursing may increase access by youth and families.
Cady, 2014 (37) Cady, 2008 (36)	To investigate the telephone interactions of advanced practice registered nurse.	University of Minnesota in USA	Children with medical complexity (*N* = 27)	Retrospective review (Descriptive study)	NA	NA	• Care coordination episodes tripled, with a significant increase (*p* < .001) between years 1 and 2. The increased episodes could explain previously reported reductions in hospitalizations for this group of children. Descriptive analysis of a program specific survey showed that parents valued having a single place to call and assistance in managing their child's complex needs.
Cormack, 2016 (38)	To improve parental experiences with healthcare delivery and collaborative health care offered at school serving children with medical complexity (CMC) by implementing telehealth services.	School-based (not specified country)	Families of the children with medical complexity attending the school (*n* = 32)	Observational study	• Process	• Measure of Processes of Care (MPOC-20)	• Parental experiences with healthcare delivery were high both before and after the implementation of telehealth at an urban public charter school for CMC. p.237
Dick, 2004 (32) Young, 2006 (44)	To describe the preferences of telehome care (THC) expressed by parents of hospitalized children and their satisfaction with this new service.	Hospital for Sick Children, Canada.	Parents with children with serious medical conditions (*n* = 10)	Before-and-after studies	• Preference • Satisfaction	• 10 cm Visual Analog Scale • Questionnaire developed for the study based on hospital satisfaction measure in Ontario interviews.	• Parents with children who have significant health care needs have a strong preference for and satisfaction with THC.
Guest, 2005 (39)	To describe how a pediatric neurology team used telemedicine to offer real help and support to families in need.	Sheffield in UK	Children with neurological impairment	Descriptive study	NA	NA	• Telemedicine help and support to families in need immediately.
Harper, 2006 (40)	To evaluate the efficacy of team-to-team interdisciplinary telemedicine evaluations.	University of Iowa Hospitals and Clinics in USA	Families who had children with chronic health care concerns and disabilities (*N* = 54)	Cross-sectional study	• Satisfaction	• 55 questions by Interviews	• Providers (multiple professionals) were equally positive about the evaluations. Data suggest that the telemedicine evaluations were viewed as good as face-to-face consultations. • Significant cost savings occurred.
Hooshmand, 2017 (35)	To provide an overview of the challenges facing children with special healthcare needs (CSHCN), telemedicine as a clinical option, and the potential of telemedicine as a bride to healthcare for CSHCN, families, and providers.	USA	Children with special health care needs	Literature review (report)	NA	NA	• Telemedicine might an effective accepted bridge between CSHCN and their providers and cost-effectiveness when considering the direct and indirect costs the families of CSCHN incure in seeking healthcare services.
Letourneau, 2003 (41)	To examine the volume of calls directed through the nursing line, to describe who calls and for which reasons, and to determine whether there is a subpopulation of high-need callers who make longer calls.	The Neurology Outpatient clinic at the Hospital for Sick Children in Toronto, Canada.	Children with epilepsy (63.5%), developmental delay (45.2%), “other” (27.4%), and headache (8.2%).	Cross-sectional study	• Volume of calls	• Demographic data collected from telephone documentation sheets.	• There is a high demand for the neurology nursing line in their clinic. Most telephone calls and most long telephone calls concerned patients with epilepsy. Nurses managed more than half of all telephone calls without physician assistance. Use of a nursing line can aid in the provision of care to complicated subspecialty patients.
Takizawa, 2011 (42)	To describe the new THC system for the serious illness children at home are developed by the authors using information and communication technologies.	Not specified. However, setting might be in Japan	Children with serious sick	Observational study	NA	NA	• THC might improve the environment for promoting home care.
Toly, 2022 (43)	To test the efficacy of a resourcefulness training© intervention designed for parent caregivers of technology-dependent children.	Not specified.	Parents caregivers with technology-dependent children (*n* = 93)	Randomized controlled trial	• Child's functional status • Type of medical Technology • Depression • Resourcefulness • Family functioning • General mental and physical health • Depressive Cognitions	• 14 item Functional Status Ⅱ-Revised (FS Ⅱ-R) • Technology Dependency Questionnaire • 20-item Center for Epidemiological Studies - Depression Scale (CES-D) • Resourcefulness Scale© • Feetham Family Functioning Survey (FFFS) • Medical Outcomes Short Form Health Survey (SF-12) • 8-item Depressive Cognitions Scale (DCS)	• Intervention improved fewer depressive cognitions and physical health compared with the attention control participants.

CES-D, Center for Epidemiological Studies—Depression Scale; CSHCN, children with special healthcare needs; CMC, children with medical complexity; DOS, depressive cognitions scale; FFFS, feetham family functioning survey; FS Ⅱ-R, functional status Ⅱ-revised; NA, not applicable; SF-12, short form health survey; THC, telehome care; UK, United Kingdom; USA, United States of America.

Overall, the disabilities included studies reported that telecommunications interventions may be helpful for children with and their families. Bennett et al. summarized studies on the treatment of obesity among youths with intellectual and developmental disabilities and pointed out the potentially significant benefit of telenursing is to maintain the continuum of care under the same treatment team, while alleviating stressors associated with travel to the treatment location (34). Cady et al. reported that care coordination episodes with advanced practice regstered nurses increased and reduced hospitalization of children with medical complexities (37). Cormack et al. reported that the utilization of telehealth in schools was enhance the family's perceptions of the health services being provided to their children with medical complexity (38). Dick et al. provided telehomecare (THC) to children with subacute healthcare needs and identified that preferences and satisfaction were strongly enhanced by use of THC (32). Guest et al. initiated a project that supported families caring for neurologically-impaired children using a real-time video link and planned to evaluate the project (39). They anticipate assurances of immediate online access to care for children and families, and the ability for care providers to connect widely and share knowledge. Harper evaluated the effectiveness of interdisciplinary telemedicine for children with special needs, which proved to be as effective as face-to-face consultations (40). Hooshmand et al. summarized studies on telemedicine for children with special healthcare needs and their families in the USA and suggested that there are significant differences across all other family cost valuables, including travel miles, cost of travel, missed work hours, wages lost, child care cost, loading cost, other costs, and total family cost between telemedicine vs. telemedicine not available (35). Letourneau et al. investigated the utilization of telephone nursing lines in neurological clinics and found that nurses could manage over 50% of all calls without physician assistance because of the high utilization of neurological nursing lines (41). Also, telenursing enhance the reassurance of patients. Takizawa et al. developed and described a telehomecare system for children with disabilities at home (42). This system helped daily caregiving and increasing feeling of peace of mind for family carers. Toly et al. evaluated the impact of a resourcefulness intervention on the mental and physical health and family functioning of parents of children requiring lifesaving technologies. The intervention was found to decrease parents’ depressive cognition and improve physical health compared to the attention control group (43).

### 4.3 Synthesis of results

#### 4.3.1 Characteristics of the telecommunications technology interventions

The characteristics of the telecommunications technology interventions are shown in Table 2. Although the studies used telecommunication technology for children with disabilities and their families, the types of telecommunication technology differed and included consultation, healthcare provision, monitoring, and education. Four studies focused on telecommunications technology in consulting with healthcare professionals. Cady et al. provided the program and two advanced practice registered nurses (APRN) interacted with families of children with medical complexity (CMC) by telephone (37). Guest et al. provided telemedicine to consult a specialist nurse or consultant through a real-time video link (39). Harper provided team-to-team interdisciplinary telemedicine projects (40). Letourneau et al. provided a nursing line in a clinic to address parental needs (41).

**Table 2:** Characteristics of telecommunications.

Author, year	Type	Content	Duration	Provider	Nurse's description	Assessment tool	Procedure	Data protection of patients	Trigger points
Cady, 2014 (37) Cady, 2008 (36)	Telephone interaction to do care coordination.	Parental follow-up, education, and support, acute and chronic condition management, care coordination with providers, care coordination with community resources, scheduling, and inpatient communication/discharge planning.	3-year study periods	The U Special Kids (USK) team members.	advanced practice registered nurse (APRN) conducted all care coordination.	Not specified.	APRN interactions with the families of children with medical complexity were conducted by telephone.	Stored data on a secured, dedicated server. Assurance that the camera is switched on and operated only by the patient or a family member. Appropriate ports and firewalls. Limiting user for the system.	Not specified.
Cormack, 2016 (38)	School based telehealth services (healthcare delivery and collaborative health care).	Access to primary care and multiple specialty providers, and collaboration of care.	8 weeks study periods	Not specified.	Nurses at the telehealth center visited and provided care. Registered nurse, and pediatric nurse practitioner at the school installed the digital otoscope and exam camera at their office.	Not specified.	Not specified.	Accessed by professionals from workplace computers.	Not specified.
Dick, 2004 (32) Young, 2006 (44)	Tele-HomeCare (THC)	Community-based home care and hospital-based services using vital signs monitors and two-way video-conferencing.	6 weeks study periods	Hospital care providers and community care providers.	Nurses at the monitoring center provided schedule and on-demand support 24 h/day, 7 days a week.	Not specified.	Not specified.	Not specified.	Not specified.
Guest, 2005 (39)	Telephone consultation	Consultation and contact to the neurology support nurse by telephone.	Not specified.	Neurology team.	The neurology support nurse support to offer help with children and families by telephone.	Not specified.	On the first day of contact the family was able to have a face-to-face consultation. And then, they can consultation with consultant and they can contact with specialist nurse who would probably have to make a home visit to assess the situation.	Not specified.	Not specified.
Harper, 2006 (40)	Five major clinical telemedicine projects.	(a) Pediatric Echo Network; (b) Emergency Department Support for Vascular Ischemia; (c) Tele Psychiatry Consultation; (d) Diabetes Education (e) Specialized Interdisciplinary Consultations.	Not specified.	Center for Disabilities and Development (CDD) team, including physicians, nurses, social workers, educational specialists, and others.	Not specified.	Not specified.	Consultation service	Clarification of who is responsible for confidentiality. Obtaining parental consent. Maintaining records of specific sessions.	Not specified.
Letourneau, 2003 (41)	The nursing line in the clinic	The content for calls were administrative issues, new changing, or worsening symptoms, test results, and others.	Telephone time was from a few seconds to 15 min.	4 clinic nurses and 13 staff neurologists.	Answering telephone calls from parents, physicians, pharmacies, and allied health care workers.	Not specified.	Telephone calls from patients and other healthcare workers.	Not specified.	Not specified.
Takizawa, 2011 (42)	Monitoring using tele-home care	Biological information from at-home patients undergoing monitoring.	Not specified.	Facility doctors, therapists, community nurses, visiting nurses.	Communication with patient's family.	Not specified.	Web devices linked to the Multi-dimensional visual communication system (MVCS) set at facilities and support home care.	Not specified.	Not specified.
Toly, 2022 (43)	Resourcefulness training©	Designed to improve on physical health and family functioning for parent caregivers of technology-dependent children.	weekly phone call (4 weeks) and resourcefulness training boosters (two and four months postintervention)	Graduate student intervention nurse	The first two authors used a systematic procedure to train the graduate student intervention nurse on the study protocol and monitor standardized administration of the resourcefulness training© intervention. Graduate student intervention nurse provided the resourcefulness training©.	Intervention checklist	Resourcefulness training was provided individually instruction and three social resourcefulness skills. In addition, instructions regarding daily log writing for four weeks to describe resourcefulness skill application in their daily life and access to the study website.	Conducted in private places.	Not specified.

Two studies focused on telecommunications technology interventions for providing healthcare. Cormack et al. provided school-based telehealth services to improve parental experiences with healthcare delivery at a school serving CMC (38). Dick et al. provided telehomecare (THC) after discharge from a tertiary-care pediatric hospital (32).

There was one study each on telecommunications technology interventions in monitoring and education. Takizawa et al. developed a telehomecare system to monitor patients’ SpO_2_ and blood pressure (42). Toly et al. provided a resourcefulness training intervention for parent caregivers of technology-dependent children regarding parents’ mental and physical health and family functioning (43).

There was various content in the telecommunications interventions. Cady et al. reported several aspects of inpatient communication/discharge planning (37). Cormack et al. provided telehealth services to enhance access to primary care and multiple specialty providers, and collaboration of care between healthcare professionals, teachers, and therapy teams at school (38). Dick et al. provided telehomecare to improve transition home after discharge for CMC by visiting home care services via videoconferencing (32). Guest et al. also provided telemedicine to neurology support nurse (39). Harper reported two-way video conferencing for children with special health and behavioral needs in rural area (40). Letourmeau et al. used a nursing line for administrative issues (41). Takizawa et al. monitored the biological information (42). Toly et al. designed a study to improve physical health and family functioning at post-intervention for parent caregivers of technology-dependent children (43).

Few studies have examined the duration of telecommunications technology interventions. Three projects have been offered since the 1990s (32, 37, 40) and one has been offered since 2003 (39). The study period ranged from four weeks to three years. Most interventions are provided by a team of healthcare professionals (32, 37, 39–42). In some studies, professionally qualified nurses, including APRN and primary care nurse practitioners, were involved in telecommunications technology interventions (37, 38). Graduate students provide resourcefulness training interventions (43). Cormack et al. did not report the provider (38). The main roles of the nurses were consultation and care coordination. Guest et al. reported that neurology support nurses offer help to children and families by telephone (39). In a study by Cormack et al. a pediatric primary care nurse practitioner visited and provided care at 12 schools (38). Clinic nurses consulted with parents, physicians, pharmacies, and other healthcare workers in the study, Letourneau et al. (41). Takizawa et al. reported that nurses consulted children with serious illnesses (42, 43). In two studies, the nurses coordinated their care schedules. Care coordination was performed by two U Special Kids (USK) APRNs (37). In one study, the nurses were scheduled to provide on-demand support (43). Harper did not report nurses’ descriptions (40). Toly et al. used an intervention checklist to conduct a resource fullness training intervention (43). Other studies did not describe the tools used for assessment.

In Cady et al., all communication with the APRN and CMC families was conducted by telephone, except for annual clinic visits (37). Guest et al. reported that, on the initial day of contact, families with children with serious neurological impairments were afforded an opportunity for in-person consultations by specialist nurses (39). Harper describes the development of a series of steps to review referrals, verify status, and ensure the availability of local professionals at telecommunications and local sites (40). Letourneau et al., nurses stayed at the neurology outpatient clinic in the hospital and answered telephone calls from parents, physicians, pharmacies, and allied healthcare workers (41). Takizawa et al. installed web terminals linked to a multidimensional visual communication system (MVCS), which are used daily to support home care and education (42). Toly et al. provided resourcefulness training. Interventionist delivered 50-minute face-to-face instruction on three social resourcefulness skills (43). Two studies did not describe procedures for telecommunications technology interventions (32, 38).

Although, no study described trigger points, Letourneau et al. (41) reported that nurses notified physicians of the telephone call for 47.1% (95% confidence interval (CI): 40.2–54.1) of index calls. As for data protection, ICT equipment was described in terms of how to access and operate it and clarifying who is responsible for data protection.

#### 4.3.2 Characteristics of children with disabilities

The characteristics of children with disabilities are shown in Table 3. Five studies focused on children with medical complexities (32, 37, 38, 42, 43) and two on children with neurodevelopmental disabilities, including developmental delays (39, 41). Harper included children with various disabilities, including medical complexity and developmental disabilities (40).

**Table 3:** Characteristics of children with disabilities.

Author, year	Population	Age	Description of the disability	Comorbidities	Types of school or nursery	Use of social services	Family situations
Children with medical complexity							
Cady, 2014 (37) Cady, 2008 (36)	Children with medical complexity.	Mean age: 7 years old	Not specified.	Genetic syndrome/congenital anomaly, followed by other conditions, cerebral palsy, neurodegenerative disease, immunodeficiency, and gastrointestinal	Not specified.	Not specified.	Not specified.
Cormack, 2016 (38)	Children with medical complexity.	4–18 years	Seizure disorders, Vision and Hearing Impairments, Intellectual disabilities, Reliance on feeding tubes, and Wheelchair bound.	Not specified.	Not specified.	Not specified.	Not specified.
Dick, 2004 (32) Young, 2006 (44)	Children had potentially life-threatening health conditions and required continued clinical support and vital signs assessment several times each day.	Most of children were less than 1 year of age.	Encephalitis, respiratory insufficiency requiring mechanical ventilation, and complex congenital heart conditions.	Not specified.	Not specified.	Not specified.	Educational status: 2/3 of the parents had exposure to college or university education.
Takizawa, 2011 (42)	Children with serious illness	Not specified.	Not specified.	Not specified.	Not specified.	Not specified.	Not specified.
Toly, 2022 (43)	Technology-dependent children based on the Office of Technology Assessment (OTA, 1987).	Median age: 63.5 months	Not specified.	Not specified.	Not specified.	Not specified.	Median age: 38 years; Marital status: 55% were married; Race: non-Hispanic: 63.4%; Higher education: partial college degree: 64.5%
Children with neurodevelopmental disabilities							
Guest, 2005 (39)	Children who suffer from serious neurological impairment.	Not specified.	Not specified.	Not specified.	Not specified.	Not specified.	Not specified.
Letourneau, 2003 (41)	Children with neurological disorders	1–7 years of age and 12 to 18 years of age (32.8%).	Epilepsy (63.5%), developmental delay (45.2%), “other” (27.4%), and headache (8.2%).	Not specified.	Not specified.	Not specified.	Not specified.
Children with various types of disabilities							
Harper, 2006 (40)	Children with chronic health care concerns and disabilities.	Child's mean age (SD), 7.5 (5.4)	Special health care needs, severe behavior disorder, swallowing disorder, and high need assistive technology.	Not specified.	Not specified.	Not specified.	Not specified.

OTA, office of technology assessment; SD, standardized deviation

Two studies included children with a mean age of approximately seven years (37, 40). Cormack et al. targeted children aged four to 18 (38). Dick et al. mainly included children under one year of age (32). Letourneau et al. focused on children one to seven years (33.9%) and 12–18 years of age (32.8%) (41). Toly et al. targeted children younger than 17 (43). Two studies did not specify the number of years (39, 42).

Four studies reported descriptions of disabilities. Cormack et al. reported that eighty percent of participants were medically fragile and with complex conditions (38). Dick et al. included respiratory insufficiency requiring mechanical ventilation, and complex congenital heart conditions (32). Harper included children with special healthcare needs (40). Letourneau et al. included children with epilepsy and developmental delay (41). Cady et al. included children with genetic syndromes/congenital anomalies and cerebral palsy (37). In addition, no studies have reported on the use of social services. Two studies reported on family situations (32, 43).

## 5 Discussion

### 5.1 Summary of evidence

Telehealth has been linked to current trends such as the transformation of traditional healthcare into accessible and cost-effective telehealth, the expansion of acute care to chronic care, and the transformation of telehealth from hospitals to homes and mobile devices; it is also becoming widely used (45). This scoping review identified 11 primary studies published between 2003 and 2020 that reported on telecommunications technology interventions involving nurses. Our findings indicate a paucity of research focusing on the implementation of telecommunications for children with disabilities and their families. Globally, 52.9 million children younger than 5 with developmental disabilities in 2016, and the global burden related to the Sustainable Developmental Goals of developmental disabilities has not improved since 1990 (46). Most included studies focused on children with medical complexities; there is a lack of studies focusing on children with neurodevelopmental disabilities.

Although we focused on telenursing, we found only one trial that addressed telenursing and included telecommunication technology interventions involving nurses. There are various synonyms for telecommunications, and trials mainly used “telehealth” and “telemedicine.” Telehealth and telemedicine were distinguished primarily by administration, with no differences in their intervention details. Telehealth is broader than telemedicine and involves electronic and telecommunication technologie (47). The Health Resources and Services Administration defines “telehealth” as a method to “support and promote long-distance clinical health care, patient and professional health-related education, and public health and administration (48).” The Centers for Medicare & Medicaid Services defines “telemedicine” as a “two-way, real time interactive communication between the patient and the physician or practitioner at [a] distant site (49).” Telenursing is defined as “the use of telecommunications technology in nursing, including the use of electromagnetic channels to transmit voice, data, and video communication signals to enhance patient care” (19). Telehealth, telemedicine, and telenursing are technological interventions for interactive communication among healthcare professionals, patients, and families. However, “telenursing” was used when the providers were limited to nurses. Most studies provided telecommunication technology interventions by teams, including nurses, which they called telehealth and telemedicine, rather than telenursing.

Telecommunications technology intervention comprises various clinical areas, such as telemental health, telerehabilitation, teledermatology, teleconsultation, and others (41). Although this study also identified various types of telecommunications technology interventions, including consultation, healthcare provision, monitoring, and education, the clinical area differed from the results of a previous study. It is possible that some clinical areas, such as mental health and rehabilitation, were provided by healthcare professionals other than nurses. Most included studies provided telecommunications technology interventions by teams, including nurses. Although we conducted a scoping review that focused on telenursing, only one study discussed it. Furthermore, although interprofessional collaboration is still developing and there is insufficient evidence to draw clear conclusions on the effects of interprofessional collaboration interventions, they might improve professional practice and healthcare outcomes (50). The results of this study suggest the need for inter-professional collaboration, even in telecommunications technology interventions settings, to provide better care for children with disabilities and their families. In the care of children with disabilities involving multiple professions, it is easy for the location of the responsible party to become unclear, and difficulties are anticipated in establishing systems and structures that ensure thorough information protection. Therefore, it is important to be sure to include in the telenursing procedure a clear determination of who is responsible for information protection, depending on the situation of the disabled child.

Telecommunications technology interventions differ in purpose and content, resulting in varying outcomes and measures. However, studies showed that telecommunications technology interventions might be feasible and could improve the care of children with disabilities and their families. Children with disabilities have barriers in accessing medical care services from healthcare professionals; telecommunication technology interventions might improve the health and experiences of care and quality of life among children with disabilities and their families (51).

### 5.2 Recommendations for our future studies

What are the recommended telenursing protocols for disabled children and their families in Japan?

In Japan, home telemedicine for children with disabilities is becoming institutionalized; however, telenursing for children with disabilities has not fully expanded. In this study, there were several reports of telenursing not only by nurses but also in collaboration with multiple professions; we believe that it is important to clarify the role of collaboration with multiple professions or nursing.

In addition, many reports have indicated that the target audience for telenursing is the family, while taking the child's situation into consideration. This suggests that, even when providing telenursing only to children, it is necessary to consider sharing information with families according to their age and intervening with them simultaneously. Due to policies to promote home care of children with disabilities, the number of children with disabilities living at home is increasing (7). As the number of children living at home has increased, there is inadequate assurance of the quantity and quality of social resources available to children with various disabilities (9, 10, 42). This situation has resulted in a lack of social resources, especially in rural areas (52). Therefore, it is necessary to expand telenursing, which is cost-effective and more accessible, and to make it available throughout the country.

Furthermore, the number of children with developmental disabilities who need support is increasing (53), and medical support in cooperation with the home and educational settings is required. Thus, support for children with disabilities in diverse situations, especially those corresponding to their living conditions, is required, and support by nurses is considered to be effective. The number of children who do not attend school has increased because of the COVID-19 pandemic, and the number of suicides among children, especially adolescents, has reached an all-time high (54). The highest proportion of clinical-level problems was observed in school-aged children with developmental disorders during the COVID-19 pandemic (55). These findings suggest the need for further development of continuous care for school-aged children and their family with developmental disorders.

Verification of telenursing for children with disabilities is lacking, and specific methods need to be developed according to the characteristics of the target population.

In this study, studies in which the target population of telenursing was families were reported. This suggests that telenursing can provide family care. Based on the above, the feature of telenursing's ability to provide continuous support that includes family members is expected to enhance the quality of life of school-aged children with developmental disorders.

### 5.3 Strength and limitations

This scoping review was rigorously conducted following the JBI methodology, and the findings were reported according to PRISMA-ScR. However, it has some limitations. First, the findings were based on limited resources. Despite being important for telehealth practice, we could not display some content such as assessment tools, trigger points, types of schools or nurseries, use of social services, and family situations because the included studies did not describe them. Second, to make our review more feasible, we only included studies reported in English and Japanese. Thus, this scoping review may not apply to relevant studies written in other languages. Third, this review aimed to map existing evidence to show a gap, and a risk of bias assessment was not performed. The quality of the included studies was unclear, and our findings should be interpreted cautiously. Consideration should also be given to the fact that our study includes results from studies conducted early after COVID-19, but may not include results reflecting on subsequent developments in telehealth.

### 5.4 Conclusion

In Japan, policies to promote home care for handicapped children are advancing and the number of handicapped children at home is increasing but social resources are in short supply, especially in rural areas. It consequently has a need to expand telenursing, which is cost-effective and readily accessible. This scoping review aimed to summarize the published literature on telenursing for children with disabilities in home care settings. Available evidence indicates a lack of research focusing on the implementation of telecommunications for children with disabilities and their families. Children's disabilities vary widely, and it is unclear for disability status of children for whom telenursing is effective. Also, even with telenursing methods, appopriate duration, effective online tools, and trigger points were not clear. Hence, further research is required to assess the effects of telecommunications technology interventions. Additionally, they should provide important information for implementing telecommunication technology safety.

## Data Availability

The raw data supporting the conclusions of this article will be made available by the authors, without undue reservation.

## Appendix 1

### Pubmed

(("Telenursing"[Mesh] OR "Telenursing"[tw] OR "Telemonitoring"[tw] OR "Telementoring"[tw] OR "Telemedicine"[tw] OR "Mobile Health"[tw] OR "mHealth"[tw] OR "Telehealth"[tw] OR "eHealth"[tw] OR "Tele nursing"[tw] OR "Tele monitoring"[tw] OR "Tele mentoring"[tw] OR "Tele medicine"[tw] OR "m Health"[tw] OR "Tele health"[tw] OR "e Health"[tw] OR "Telenurs*"[tw] OR "Telemonitor*"[tw] OR "Telementor*"[tw] OR "Telemed*"[tw] OR "mHealth*"[tw] OR "Telehealth*"[tw] OR "eHealth*"[tw] OR "Tele nurs*"[tw] OR "Tele monitor*"[tw] OR "Tele mentor*"[tw] OR "Tele medic*"[tw] OR "m Health*"[tw] OR "Tele health*"[tw] OR "e Health*"[tw] OR "tele mental health"[tw] OR "tele p"[tw] OR (["Delivery of Health Care"[Mesh] OR "Health Services Accessibility"[Mesh]] AND ("Internet"[mesh] OR "internet"[tw] OR "internet*"[tw]))) AND ("Developmental Disabilities"[Mesh] OR "Developmental Disabilities"[tw] OR "Developmental disability"[tw] OR "Child Development Disorder"[tw] OR "Child Development Disorders"[tw] OR "Developmental Delay Disorders"[tw] OR "Developmental Delay Disorder"[tw] OR "Developmental Delay"[tw] OR "Child Development Deviations"[tw] OR "Child Development Deviation"[tw] OR "Autism Spectrum Disorder"[Mesh] OR "Autism"[tw] OR "autistic"[tw] OR "asperger"[tw] OR "asperger*"[tw] OR "Attention Deficit Disorder with Hyperactivity"[Mesh] OR "ADDH"[tw] OR "ADHD"[tw] OR "Attention Deficit Disorder"[tw] OR "Attention Deficit Hyperactivity "[tw] OR "Hyperkinetic Syndrome"[tw] OR "Minimal Brain Dysfunction"[tw] OR "Learning Disabilities"[Mesh] OR "Learning Disabilities"[tw] OR "Learning disability"[tw] OR "Dyscalculia"[tw] OR "Dyslexia"[tw] OR "Learning Disorder"[tw] OR "Learning Disorders"[tw] OR "Intellectual Disability"[Mesh] OR "Disability"[tw] OR "Disabilities"[tw] OR "disab*"[tw] OR "special needs"[tw] OR "special need"[tw] OR "handicap"[tw] OR "handicaps"[tw] OR "handicapped"[tw] OR "handicap*"[tw] OR "impairment"[tw] OR "impairments"[tw] OR "impaired*"[tw] OR "impair*"[tw] OR "Cognitive Dysfunction"[Mesh] OR "Cognitive Dysfunction"[tw] OR "Cognitive Dysfunction*"[tw] OR "Neurodevelopmental Disorders"[Mesh] OR "Neurodevelopmental Disorders"[tw] OR "neurodevelopmental disorder"[tw] OR "Mental Health"[mesh] OR "mental health"[tw] OR "Emotions"[Mesh] OR "Emotions"[tw] OR "Emotion*"[tw] OR "depression"[tw] OR "depress*"[tw] OR "anxiety"[tw] OR "anxiet*"[tw]) AND ("Caregivers"[Mesh] OR "Caregivers"[tw] OR "Caregiver"[tw] OR "caregiv*"[tw] OR "carer"[tw] OR "carers"[tw] OR "Parents"[Mesh] OR "Parents"[tw] OR "Parent"[tw] OR "parent*"[tw] OR "Family"[Mesh] OR "family"[tw] OR "families"[tw] OR "mother"[tw] OR "mothers"[tw] OR "mother*"[tw] OR "father"[tw] OR "fathers"[tw] OR "father*"[tw] OR "sibling"[tw] OR "sibling"[tw] OR "sibling*"[tw] OR "Sisters"[tw] OR "Sister"[tw] OR "Brothers"[tw] OR "Brother"[tw] OR "Home Care Services"[Mesh] OR "home care"[tw]) AND ("Child"[Mesh] OR "child"[tw] OR "children"[tw] OR "Infant"[Mesh] OR "infant"[tw] OR "infants"[tw] OR "infancy"[tw] OR "newborn"[tw] OR "newborns"[tw] OR "new-born"[tw] OR "new-borns"[tw] OR "neonate"[tw] OR "neonates"[tw] OR "neonatal"[tw] OR "neo-nate"[tw] OR "neo-nates"[tw] OR "neo-natal"[tw] OR "neonatology"[tw] OR "NICU"[ti] OR "premature"[tw] OR "prematures"[tw] OR "pre-mature"[tw] OR "pre-matures"[tw] OR "preterm"[tw] OR "pre-term"[tw] OR "postnatal"[tw] OR "post-natal"[tw] OR "baby"[tw] OR "babies"[tw] OR "suckling"[tw] OR "sucklings"[tw] OR "toddler"[tw] OR "toddlers"[tw] OR "childhood"[tw] OR "schoolchild"[tw] OR "schoolchildren"[tw] OR "childcare"[tw] OR "child-care"[tw] OR "young"[ti] OR "youngster"[tw] OR "youngsters"[tw] OR "preschool"[tw] OR "pre-school"[tw] OR "kid"[tw] OR "kids"[tw] OR "boy"[tw] OR "boys"[tw] OR "girl"[tw] OR "girls"[tw] OR "Adolescent"[Mesh] OR "adolescent"[tw] OR "adolescents"[tw] OR "adolescence"[tw] OR "pre-adolescent"[tw] OR "pre-adolescents"[tw] OR "pre-adolescence"[tw] OR "schoolage"[tw] OR "schoolboy"[tw] OR "schoolboys"[tw] OR "schoolgirl"[tw] OR "schoolgirls"[tw] OR "pre-puber"[tw] OR "pre-pubers"[tw] OR "pre-puberty"[tw] OR "prepuber"[tw] OR "prepubers"[tw] OR "prepuberty"[tw] OR "puber"[tw] OR "pubers"[tw] OR "puberty"[tw] OR "puberal"[tw] OR "teenager"[tw] OR "teenagers"[tw] OR "teens"[tw] OR "youth"[tw] OR "youths"[tw] OR "underaged"[tw] OR "under-aged"[tw] OR "Pediatrics"[Mesh] OR "Pediatric"[tw] OR "Pediatrics"[tw] OR "Paediatric"[tw] OR "Paediatrics"[tw] OR "PICU"[ti] OR ["child"[all fields] NOT child[au]] OR children*[all fields] OR schoolchild*[all fields] OR "infant"[all fields] OR "infants"[all fields] OR "infancy"[all fields] OR adolesc*[all fields] OR pediat*[all fields] OR paediat*[all fields] OR neonat*[all fields] OR toddler*[all fields] OR "teen"[all fields] OR "teens"[all fields] OR teenager*[all fields] OR preteen*[all fields] OR newborn*[all fields] OR postneonat*[all fields] OR postnatal*[all fields] OR "puberty"[all fields] OR preschool*[all fields] OR suckling*[all fields] OR "juvenile"[all fields] OR "new born"[all fields] OR "new borns"[all fields] OR new-born*[all fields] OR neo-nat*[all fields] OR neonat*[all fields] OR perinat*[all fields] OR underag*[all fields] OR "under age"[all fields] OR "under aged"[all fields] OR youth*[all fields] OR kinder*[all fields] OR pubescen*[all fields] OR prepubescen*[all fields] OR "prepuberty"[all fields] OR "school age"[all fields] OR "schoolage"[all fields] OR "school ages"[all fields] OR schoolage*[all fields] OR "one year old"[ti] OR "two year old"[ti] OR "three year old"[ti] OR "four year old"[ti] OR "five year old"[ti] OR "six year old"[ti] OR "seven year old"[ti] OR "eight year old"[ti] OR "nine year old"[ti] OR "ten year old"[ti] OR "eleven year old"[ti] OR "twelve year old"[ti] OR "thirteen year old"[ti] OR "fourteen year old"[ti] OR "fifteen year old"[ti] OR "sixteen year old"[ti] OR "seventeen year old"[ti] OR "eighteen year old"[ti] OR "1 year old"[ti] OR "2 year old"[ti] OR "3 year old"[ti] OR "4 year old"[ti] OR "5 year old"[ti] OR "6 year old"[ti] OR "7 year old"[ti] OR "8 year old"[ti] OR "9 year old"[ti] OR "10 year old"[ti] OR "11 year old"[ti] OR "12 year old"[ti] OR "13 year old"[ti] OR "14 year old"[ti] OR "15 year old"[ti] OR "16 year old"[ti] OR "17 year old"[ti] OR "18 year old"[ti] OR "two years old"[ti] OR "three years old"[ti] OR "four years old"[ti] OR "five years old"[ti] OR "six years old"[ti] OR "seven years old"[ti] OR "eight years old"[ti] OR "nine years old"[ti] OR "ten years old"[ti] OR "eleven years old"[ti] OR "twelve years old"[ti] OR "thirteen years old"[ti] OR "fourteen years old"[ti] OR "fifteen years old"[ti] OR "sixteen years old"[ti] OR "seventeen years old"[ti] OR "eighteen years old"[ti] OR "2 years old"[ti] OR "3 years old"[ti] OR "4 years old"[ti] OR "5 years old"[ti] OR "6 years old"[ti] OR "7 years old"[ti] OR "8 years old"[ti] OR "9 years old"[ti] OR "10 years old"[ti] OR "11 years old"[ti] OR "12 years old"[ti] OR "13 years old"[ti] OR "14 years old"[ti] OR "15 years old"[ti] OR "16 years old"[ti] OR "17 years old"[ti] OR "18 years old"[ti]) AND [japanese[la] OR english[la]] AND ["1990/01/01"[PDAT]: "3000/12/31"[PDAT]])

### Embase (OVID)

((exp "Telenursing" OR exp "Telemedicine" OR exp "Telehealth" OR "Telenursing".mp OR "Telemonitoring".mp OR "Telementoring".mp OR "Telemedicine".mp OR "Mobile Health".mp OR "mHealth".mp OR "Telehealth".mp OR "eHealth".mp OR "Tele nursing".mp OR "Tele monitoring".mp OR "Tele mentoring".mp OR "Tele medicine".mp OR "m Health".mp OR "Tele health".mp OR "e Health".mp OR "Telenurs*".mp OR "Telemonitor*".mp OR "Telementor*".mp OR "Telemed*".mp OR "mHealth*".mp OR "Telehealth*".mp OR "eHealth*".mp OR "Tele nurs*".mp OR "Tele monitor*".mp OR "Tele mentor*".mp OR "Tele medic*".mp OR "m Health*".mp OR "Tele health*".mp OR "e Health*".mp OR "tele mental health".mp OR "tele p".mp OR [(exp "Health Care Delivery" OR exp "Health Services Access"/) AND (exp "Internet" OR "internet".mp OR "internet*".mp)]) AND (exp "Developmental Disorder" OR "Developmental Disabilities".mp OR "Developmental disability".mp OR "Child Development Disorder".mp OR "Child Development Disorders".mp OR "Developmental Delay Disorders".mp OR "Developmental Delay Disorder".mp OR "Developmental Delay".mp OR "Child Development Deviations".mp OR "Child Development Deviation".mp OR exp "Autism" OR "Autism".mp OR "autistic".mp OR "asperger".mp OR "asperger*".mp OR exp "Attention Deficit Disorder" OR "ADDH".mp OR "ADHD".mp OR "Attention Deficit Disorder".mp OR "Attention Deficit Hyperactivity ".mp OR "Hyperkinetic Syndrome".mp OR "Minimal Brain Dysfunction".mp OR exp "Learning Disorder" OR "Learning Disabilities".mp OR "Learning disability".mp OR "Dyscalculia".mp OR "Dyslexia".mp OR "Learning Disorder".mp OR "Learning Disorders".mp OR exp "Intellectual Impairment" OR "Disability".mp OR "Disabilities".mp OR "disab*".mp OR "special needs".mp OR "special need".mp OR "handicap".mp OR "handicaps".mp OR "handicapped".mp OR "handicap*".mp OR "impairment".mp OR "impairments".mp OR "impaired*".mp OR "impair*".mp OR exp "Cognitive Defect" OR "Cognitive Dysfunction".mp OR "Cognitive Dysfunction*".mp OR "Neurodevelopmental Disorders".mp OR "neurodevelopmental disorder".mp OR exp "Mental Health" OR "mental health".mp OR exp "Emotion" OR "Emotions".mp OR "Emotion*".mp OR "depression".mp OR "depress*".mp OR "anxiety".mp OR "anxiet*".mp) AND (exp "Caregiver" OR "Caregivers".mp OR "Caregiver".mp OR "caregiv*".mp OR "carer".mp OR "carers".mp OR exp "Parent" OR "Parents".mp OR "Parent".mp OR "parent*".mp OR exp "Family" OR "family".mp OR "families".mp OR "mother".mp OR "mothers".mp OR "mother*".mp OR "father".mp OR "fathers".mp OR "father*".mp OR "sibling".mp OR "sibling".mp OR "sibling*".mp OR "Sisters".mp OR "Sister".mp OR "Brothers".mp OR "Brother".mp OR exp "Home Care" OR "home care".mp) AND (exp "Child" OR "child".mp OR "children".mp OR exp "Infant" OR "infant".mp OR "infants".mp OR "infancy".mp OR "newborn".mp OR "newborns".mp OR "new-born".mp OR "new-borns".mp OR "neonate".mp OR "neonates".mp OR "neonatal".mp OR "neo-nate".mp OR "neo-nates".mp OR "neo-natal".mp OR "neonatology".mp OR "NICU".ti OR "premature".mp OR "prematures".mp OR "pre-mature".mp OR "pre-matures".mp OR "preterm".mp OR "pre-term".mp OR "postnatal".mp OR "post-natal".mp OR "baby".mp OR "babies".mp OR "suckling".mp OR "sucklings".mp OR "toddler".mp OR "toddlers".mp OR "childhood".mp OR "schoolchild".mp OR "schoolchildren".mp OR "childcare".mp OR "child-care".mp OR "young".ti OR "youngster".mp OR "youngsters".mp OR "preschool".mp OR "pre-school".mp OR "kid".mp OR "kids".mp OR "boy".mp OR "boys".mp OR "girl".mp OR "girls".mp OR exp "Adolescent" OR "adolescent".mp OR "adolescents".mp OR "adolescence".mp OR "pre-adolescent".mp OR "pre-adolescents".mp OR "pre-adolescence".mp OR "schoolage".mp OR "schoolboy".mp OR "schoolboys".mp OR "schoolgirl".mp OR "schoolgirls".mp OR "pre-puber".mp OR "pre-pubers".mp OR "pre-puberty".mp OR "prepuber".mp OR "prepubers".mp OR "prepuberty".mp OR "puber".mp OR "pubers".mp OR "puberty".mp OR "puberal".mp OR "teenager".mp OR "teenagers".mp OR "teens".mp OR "youth".mp OR "youths".mp OR "underaged".mp OR "under-aged".mp OR exp "Pediatrics" OR "Pediatric".mp OR "Pediatrics".mp OR "Paediatric".mp OR "Paediatrics".mp OR "PICU".ti OR "child".mp OR children*.mp OR schoolchild*.mp OR "infant".mp OR "infants".mp OR "infancy".mp OR adolesc*.mp OR pediat*.mp OR paediat*.mp OR neonat*.mp OR toddler*.mp OR "teen".mp OR "teens".mp OR teenager*.mp OR preteen*.mp OR newborn*.mp OR postneonat*.mp OR postnatal*.mp OR "puberty".mp OR preschool*.mp OR suckling*.mp OR "juvenile".mp OR "new born".mp OR "new borns".mp OR new-born*.mp OR neo-nat*.mp OR neonat*.mp OR perinat*.mp OR underag*.mp OR "under age".mp OR "under aged".mp OR youth*.mp OR kinder*.mp OR pubescen*.mp OR prepubescen*.mp OR "prepuberty".mp OR "school age".mp OR "schoolage".mp OR "school ages".mp OR schoolage*.mp OR "one year old".ti OR "two year old".ti OR "three year old".ti OR "four year old".ti OR "five year old".ti OR "six year old".ti OR "seven year old".ti OR "eight year old".ti OR "nine year old".ti OR "ten year old".ti OR "eleven year old".ti OR "twelve year old".ti OR "thirteen year old".ti OR "fourteen year old".ti OR "fifteen year old".ti OR "sixteen year old".ti OR "seventeen year old".ti OR "eighteen year old".ti OR "1 year old".ti OR "2 year old".ti OR "3 year old".ti OR "4 year old".ti OR "5 year old".ti OR "6 year old".ti OR "7 year old".ti OR "8 year old".ti OR "9 year old".ti OR "10 year old".ti OR "11 year old".ti OR "12 year old".ti OR "13 year old".ti OR "14 year old".ti OR "15 year old".ti OR "16 year old".ti OR "17 year old".ti OR "18 year old".ti OR "two years old".ti OR "three years old".ti OR "four years old".ti OR "five years old".ti OR "six years old".ti OR "seven years old".ti OR "eight years old".ti OR "nine years old".ti OR "ten years old".ti OR "eleven years old".ti OR "twelve years old".ti OR "thirteen years old".ti OR "fourteen years old".ti OR "fifteen years old".ti OR "sixteen years old".ti OR "seventeen years old".ti OR "eighteen years old".ti OR "2 years old".ti OR "3 years old".ti OR "4 years old".ti OR "5 years old".ti OR "6 years old".ti OR "7 years old".ti OR "8 years old".ti OR "9 years old".ti OR "10 years old".ti OR "11 years old".ti OR "12 years old".ti OR "13 years old".ti OR "14 years old".ti OR "15 years old".ti OR "16 years old".ti OR "17 years old".ti OR "18 years old".ti) AND (japanese.la OR english.la) AND 1990:2023.(sa_year))

### Cochrane library

(("Telenursing" OR "Telemedicine" OR "Telehealth" OR "Telenursing" OR "Telemonitoring" OR "Telementoring" OR "Telemedicine" OR "Mobile Health" OR "mHealth" OR "Telehealth" OR "eHealth" OR "Tele nursing" OR "Tele monitoring" OR "Tele mentoring" OR "Tele medicine" OR "m Health" OR "Tele health" OR "e Health" OR "Telenurs*" OR "Telemonitor*" OR "Telementor*" OR "Telemed*" OR "mHealth*" OR "Telehealth*" OR "eHealth*" OR "Tele nurs*" OR "Tele monitor*" OR "Tele mentor*" OR "Tele medic*" OR "m Health*" OR "Tele health*" OR "e Health*" OR "tele mental health" OR "tele p" OR [("Health Care Delivery" OR "Health Services Access") AND ("Internet" OR "internet" OR "internet*")]) AND ("Developmental Disorder" OR "Developmental Disabilities" OR "Developmental disability" OR "Child Development Disorder" OR "Child Development Disorders" OR "Developmental Delay Disorders" OR "Developmental Delay Disorder" OR "Developmental Delay" OR "Child Development Deviations" OR "Child Development Deviation" OR "Autism" OR "Autism" OR "autistic" OR "asperger" OR "asperger*" OR "Attention Deficit Disorder" OR "ADDH" OR "ADHD" OR "Attention Deficit Disorder" OR "Attention Deficit Hyperactivity " OR "Hyperkinetic Syndrome" OR "Minimal Brain Dysfunction" OR "Learning Disorder" OR "Learning Disabilities" OR "Learning disability" OR "Dyscalculia" OR "Dyslexia" OR "Learning Disorder" OR "Learning Disorders" OR "Intellectual Impairment" OR "Disability" OR "Disabilities" OR "disab*" OR "special needs" OR "special need" OR "handicap" OR "handicaps" OR "handicapped" OR "handicap*" OR "impairment" OR "impairments" OR "impaired*" OR "impair*" OR "Cognitive Defect" OR "Cognitive Dysfunction" OR "Cognitive Dysfunction*" OR "Neurodevelopmental Disorders" OR "neurodevelopmental disorder" OR "Mental Health" OR "mental health" OR "Emotion" OR "Emotions" OR "Emotion*" OR "depression" OR "depress*" OR "anxiety" OR "anxiet*") AND ("Caregiver" OR "Caregivers" OR "Caregiver" OR "caregiv*" OR "carer" OR "carers" OR "Parent" OR "Parents" OR "Parent" OR "parent*" OR "Family" OR "family" OR "families" OR "mother" OR "mothers" OR "mother*" OR "father" OR "fathers" OR "father*" OR "sibling" OR "sibling" OR "sibling*" OR "Sisters" OR "Sister" OR "Brothers" OR "Brother" OR "Home Care" OR "home care") AND ("Child" OR "child" OR "children" OR "Infant" OR "infant" OR "infants" OR "infancy" OR "newborn" OR "newborns" OR "new born" OR "new borns" OR "neonate" OR "neonates" OR "neonatal" OR "neo nate" OR "neo nates" OR "neo natal" OR "neonatology" OR "NICU" OR "premature" OR "prematures" OR "pre mature" OR "pre matures" OR "preterm" OR "pre term" OR "postnatal" OR "post natal" OR "baby" OR "babies" OR "suckling" OR "sucklings" OR "toddler" OR "toddlers" OR "childhood" OR "schoolchild" OR "schoolchildren" OR "childcare" OR "child care" OR "young" OR "youngster" OR "youngsters" OR "preschool" OR "pre school" OR "kid" OR "kids" OR "boy" OR "boys" OR "girl" OR "girls" OR "Adolescent" OR "adolescent" OR "adolescents" OR "adolescence" OR "pre adolescent" OR "pre adolescents" OR "pre adolescence" OR "schoolage" OR "schoolboy" OR "schoolboys" OR "schoolgirl" OR "schoolgirls" OR "pre puber" OR "pre pubers" OR "pre puberty" OR "prepuber" OR "prepubers" OR "prepuberty" OR "puber" OR "pubers" OR "puberty" OR "puberal" OR "teenager" OR "teenagers" OR "teens" OR "youth" OR "youths" OR "underaged" OR "under aged" OR "Pediatrics" OR "Pediatric" OR "Pediatrics" OR "Paediatric" OR "Paediatrics" OR "PICU" OR "child" OR children* OR schoolchild* OR "infant" OR "infants" OR "infancy" OR adolesc* OR pediat* OR paediat* OR neonat* OR toddler* OR "teen" OR "teens" OR teenager* OR preteen* OR newborn* OR postneonat* OR postnatal* OR "puberty" OR preschool* OR suckling* OR "juvenile" OR "new born" OR "new borns" OR new born* OR neo nat* OR neonat* OR perinat* OR underag* OR "under age" OR "under aged" OR youth* OR kinder* OR pubescen* OR prepubescen* OR "prepuberty" OR "school age" OR "schoolage" OR "school ages" OR schoolage* OR "one year old" OR "two year old" OR "three year old" OR "four year old" OR "five year old" OR "six year old" OR "seven year old" OR "eight year old" OR "nine year old" OR "ten year old" OR "eleven year old" OR "twelve year old" OR "thirteen year old" OR "fourteen year old" OR "fifteen year old" OR "sixteen year old" OR "seventeen year old" OR "eighteen year old" OR "1 year old" OR "2 year old" OR "3 year old" OR "4 year old" OR "5 year old" OR "6 year old" OR "7 year old" OR "8 year old" OR "9 year old" OR "10 year old" OR "11 year old" OR "12 year old" OR "13 year old" OR "14 year old" OR "15 year old" OR "16 year old" OR "17 year old" OR "18 year old" OR "two years old" OR "three years old" OR "four years old" OR "five years old" OR "six years old" OR "seven years old" OR "eight years old" OR "nine years old" OR "ten years old" OR "eleven years old" OR "twelve years old" OR "thirteen years old" OR "fourteen years old" OR "fifteen years old" OR "sixteen years old" OR "seventeen years old" OR "eighteen years old" OR "2 years old" OR "3 years old" OR "4 years old" OR "5 years old" OR "6 years old" OR "7 years old" OR "8 years old" OR "9 years old" OR "10 years old" OR "11 years old" OR "12 years old" OR "13 years old" OR "14 years old" OR "15 years old" OR "16 years old" OR "17 years old" OR "18 years old")):ti,ab,kw

### Emcare (OVID)

((exp "Telenursing" OR exp "Telemedicine" OR exp "Telehealth" OR "Telenursing".mp OR "Telemonitoring".mp OR "Telementoring".mp OR "Telemedicine".mp OR "Mobile Health".mp OR "mHealth".mp OR "Telehealth".mp OR "eHealth".mp OR "Tele nursing".mp OR "Tele monitoring".mp OR "Tele mentoring".mp OR "Tele medicine".mp OR "m Health".mp OR "Tele health".mp OR "e Health".mp OR "Telenurs*".mp OR "Telemonitor*".mp OR "Telementor*".mp OR "Telemed*".mp OR "mHealth*".mp OR "Telehealth*".mp OR "eHealth*".mp OR "Tele nurs*".mp OR "Tele monitor*".mp OR "Tele mentor*".mp OR "Tele medic*".mp OR "m Health*".mp OR "Tele health*".mp OR "e Health*".mp OR "tele mental health".mp OR "tele p".mp OR ((exp "Health Care Delivery" OR exp "Health Services Access"/) AND (exp "Internet" OR "internet".mp OR "internet*".mp))) AND (exp "Developmental Disorder" OR "Developmental Disabilities".mp OR "Developmental disability".mp OR "Child Development Disorder".mp OR "Child Development Disorders".mp OR "Developmental Delay Disorders".mp OR "Developmental Delay Disorder".mp OR "Developmental Delay".mp OR "Child Development Deviations".mp OR "Child Development Deviation".mp OR exp "Autism" OR "Autism".mp OR "autistic".mp OR "asperger".mp OR "asperger*".mp OR exp "Attention Deficit Disorder" OR "ADDH".mp OR "ADHD".mp OR "Attention Deficit Disorder".mp OR "Attention Deficit Hyperactivity ".mp OR "Hyperkinetic Syndrome".mp OR "Minimal Brain Dysfunction".mp OR exp "Learning Disorder" OR "Learning Disabilities".mp OR "Learning disability".mp OR "Dyscalculia".mp OR "Dyslexia".mp OR "Learning Disorder".mp OR "Learning Disorders".mp OR exp "Intellectual Impairment" OR "Disability".mp OR "Disabilities".mp OR "disab*".mp OR "special needs".mp OR "special need".mp OR "handicap".mp OR "handicaps".mp OR "handicapped".mp OR "handicap*".mp OR "impairment".mp OR "impairments".mp OR "impaired*".mp OR "impair*".mp OR exp "Cognitive Defect" OR "Cognitive Dysfunction".mp OR "Cognitive Dysfunction*".mp OR "Neurodevelopmental Disorders".mp OR "neurodevelopmental disorder".mp OR exp "Mental Health" OR "mental health".mp OR exp "Emotion" OR "Emotions".mp OR "Emotion*".mp OR "depression".mp OR "depress*".mp OR "anxiety".mp OR "anxiet*".mp) AND (exp "Caregiver" OR "Caregivers".mp OR "Caregiver".mp OR "caregiv*".mp OR "carer".mp OR "carers".mp OR exp "Parent" OR "Parents".mp OR "Parent".mp OR "parent*".mp OR exp "Family" OR "family".mp OR "families".mp OR "mother".mp OR "mothers".mp OR "mother*".mp OR "father".mp OR "fathers".mp OR "father*".mp OR "sibling".mp OR "sibling".mp OR "sibling*".mp OR "Sisters".mp OR "Sister".mp OR "Brothers".mp OR "Brother".mp OR exp "Home Care" OR "home care".mp) AND (exp "Child" OR "child".mp OR "children".mp OR exp "Infant" OR "infant".mp OR "infants".mp OR "infancy".mp OR "newborn".mp OR "newborns".mp OR "new-born".mp OR "new-borns".mp OR "neonate".mp OR "neonates".mp OR "neonatal".mp OR "neo-nate".mp OR "neo-nates".mp OR "neo-natal".mp OR "neonatology".mp OR "NICU".ti OR "premature".mp OR "prematures".mp OR "pre-mature".mp OR "pre-matures".mp OR "preterm".mp OR "pre-term".mp OR "postnatal".mp OR "post-natal".mp OR "baby".mp OR "babies".mp OR "suckling".mp OR "sucklings".mp OR "toddler".mp OR "toddlers".mp OR "childhood".mp OR "schoolchild".mp OR "schoolchildren".mp OR "childcare".mp OR "child-care".mp OR "young".ti OR "youngster".mp OR "youngsters".mp OR "preschool".mp OR "pre-school".mp OR "kid".mp OR "kids".mp OR "boy".mp OR "boys".mp OR "girl".mp OR "girls".mp OR exp "Adolescent" OR "adolescent".mp OR "adolescents".mp OR "adolescence".mp OR "pre-adolescent".mp OR "pre-adolescents".mp OR "pre-adolescence".mp OR "schoolage".mp OR "schoolboy".mp OR "schoolboys".mp OR "schoolgirl".mp OR "schoolgirls".mp OR "pre-puber".mp OR "pre-pubers".mp OR "pre-puberty".mp OR "prepuber".mp OR "prepubers".mp OR "prepuberty".mp OR "puber".mp OR "pubers".mp OR "puberty".mp OR "puberal".mp OR "teenager".mp OR "teenagers".mp OR "teens".mp OR "youth".mp OR "youths".mp OR "underaged".mp OR "under-aged".mp OR exp "Pediatrics" OR "Pediatric".mp OR "Pediatrics".mp OR "Paediatric".mp OR "Paediatrics".mp OR "PICU".ti OR "child".mp OR children*.mp OR schoolchild*.mp OR "infant".mp OR "infants".mp OR "infancy".mp OR adolesc*.mp OR pediat*.mp OR paediat*.mp OR neonat*.mp OR toddler*.mp OR "teen".mp OR "teens".mp OR teenager*.mp OR preteen*.mp OR newborn*.mp OR postneonat*.mp OR postnatal*.mp OR "puberty".mp OR preschool*.mp OR suckling*.mp OR "juvenile".mp OR "new born".mp OR "new borns".mp OR new-born*.mp OR neo-nat*.mp OR neonat*.mp OR perinat*.mp OR underag*.mp OR "under age".mp OR "under aged".mp OR youth*.mp OR kinder*.mp OR pubescen*.mp OR prepubescen*.mp OR "prepuberty".mp OR "school age".mp OR "schoolage".mp OR "school ages".mp OR schoolage*.mp OR "one year old".ti OR "two year old".ti OR "three year old".ti OR "four year old".ti OR "five year old".ti OR "six year old".ti OR "seven year old".ti OR "eight year old".ti OR "nine year old".ti OR "ten year old".ti OR "eleven year old".ti OR "twelve year old".ti OR "thirteen year old".ti OR "fourteen year old".ti OR "fifteen year old".ti OR "sixteen year old".ti OR "seventeen year old".ti OR "eighteen year old".ti OR "1 year old".ti OR "2 year old".ti OR "3 year old".ti OR "4 year old".ti OR "5 year old".ti OR "6 year old".ti OR "7 year old".ti OR "8 year old".ti OR "9 year old".ti OR "10 year old".ti OR "11 year old".ti OR "12 year old".ti OR "13 year old".ti OR "14 year old".ti OR "15 year old".ti OR "16 year old".ti OR "17 year old".ti OR "18 year old".ti OR "two years old".ti OR "three years old".ti OR "four years old".ti OR "five years old".ti OR "six years old".ti OR "seven years old".ti OR "eight years old".ti OR "nine years old".ti OR "ten years old".ti OR "eleven years old".ti OR "twelve years old".ti OR "thirteen years old".ti OR "fourteen years old".ti OR "fifteen years old".ti OR "sixteen years old".ti OR "seventeen years old".ti OR "eighteen years old".ti OR "2 years old".ti OR "3 years old".ti OR "4 years old".ti OR "5 years old".ti OR "6 years old".ti OR "7 years old".ti OR "8 years old".ti OR "9 years old".ti OR "10 years old".ti OR "11 years old".ti OR "12 years old".ti OR "13 years old".ti OR "14 years old".ti OR "15 years old".ti OR "16 years old".ti OR "17 years old".ti OR "18 years old".ti) AND (japanese.la OR english.la) AND 1990:2023.(sa_year))

### PsycINFO (EbscoHOST)

((TI("Telenursing" OR "Telemedicine" OR "Telehealth" OR "Telenursing" OR "Telemonitoring" OR "Telementoring" OR "Telemedicine" OR "Mobile Health" OR "mHealth" OR "Telehealth" OR "eHealth" OR "Tele nursing" OR "Tele monitoring" OR "Tele mentoring" OR "Tele medicine" OR "m Health" OR "Tele health" OR "e Health" OR "Telenurs*" OR "Telemonitor*" OR "Telementor*" OR "Telemed*" OR "mHealth*" OR "Telehealth*" OR "eHealth*" OR "Tele nurs*" OR "Tele monitor*" OR "Tele mentor*" OR "Tele medic*" OR "m Health*" OR "Tele health*" OR "e Health*" OR "tele mental health" OR "tele p" OR (("Health Care Delivery" OR "Health Services Access") AND ("Internet" OR "internet" OR "internet*"))) OR SU("Telenursing" OR "Telemedicine" OR "Telehealth" OR "Telenursing" OR "Telemonitoring" OR "Telementoring" OR "Telemedicine" OR "Mobile Health" OR "mHealth" OR "Telehealth" OR "eHealth" OR "Tele nursing" OR "Tele monitoring" OR "Tele mentoring" OR "Tele medicine" OR "m Health" OR "Tele health" OR "e Health" OR "Telenurs*" OR "Telemonitor*" OR "Telementor*" OR "Telemed*" OR "mHealth*" OR "Telehealth*" OR "eHealth*" OR "Tele nurs*" OR "Tele monitor*" OR "Tele mentor*" OR "Tele medic*" OR "m Health*" OR "Tele health*" OR "e Health*" OR "tele mental health" OR "tele p" OR (("Health Care Delivery" OR "Health Services Access") AND ("Internet" OR "internet" OR "internet*"))) OR MA("Telenursing" OR "Telemedicine" OR "Telehealth" OR "Telenursing" OR "Telemonitoring" OR "Telementoring" OR "Telemedicine" OR "Mobile Health" OR "mHealth" OR "Telehealth" OR "eHealth" OR "Tele nursing" OR "Tele monitoring" OR "Tele mentoring" OR "Tele medicine" OR "m Health" OR "Tele health" OR "e Health" OR "Telenurs*" OR "Telemonitor*" OR "Telementor*" OR "Telemed*" OR "mHealth*" OR "Telehealth*" OR "eHealth*" OR "Tele nurs*" OR "Tele monitor*" OR "Tele mentor*" OR "Tele medic*" OR "m Health*" OR "Tele health*" OR "e Health*" OR "tele mental health" OR "tele p" OR (("Health Care Delivery" OR "Health Services Access") AND ("Internet" OR "internet" OR "internet*"))) OR AB("Telenursing" OR "Telemedicine" OR "Telehealth" OR "Telenursing" OR "Telemonitoring" OR "Telementoring" OR "Telemedicine" OR "Mobile Health" OR "mHealth" OR "Telehealth" OR "eHealth" OR "Tele nursing" OR "Tele monitoring" OR "Tele mentoring" OR "Tele medicine" OR "m Health" OR "Tele health" OR "e Health" OR "Telenurs*" OR "Telemonitor*" OR "Telementor*" OR "Telemed*" OR "mHealth*" OR "Telehealth*" OR "eHealth*" OR "Tele nurs*" OR "Tele monitor*" OR "Tele mentor*" OR "Tele medic*" OR "m Health*" OR "Tele health*" OR "e Health*" OR "tele mental health" OR "tele p" OR (("Health Care Delivery" OR "Health Services Access") AND ("Internet" OR "internet" OR "internet*")))) AND (TI("Developmental Disorder" OR "Developmental Disabilities" OR "Developmental disability" OR "Child Development Disorder" OR "Child Development Disorders" OR "Developmental Delay Disorders" OR "Developmental Delay Disorder" OR "Developmental Delay" OR "Child Development Deviations" OR "Child Development Deviation" OR "Autism" OR "Autism" OR "autistic" OR "asperger" OR "asperger*" OR "Attention Deficit Disorder" OR "ADDH" OR "ADHD" OR "Attention Deficit Disorder" OR "Attention Deficit Hyperactivity " OR "Hyperkinetic Syndrome" OR "Minimal Brain Dysfunction" OR "Learning Disorder" OR "Learning Disabilities" OR "Learning disability" OR "Dyscalculia" OR "Dyslexia" OR "Learning Disorder" OR "Learning Disorders" OR "Intellectual Impairment" OR "Disability" OR "Disabilities" OR "disab*" OR "special needs" OR "special need" OR "handicap" OR "handicaps" OR "handicapped" OR "handicap*" OR "impairment" OR "impairments" OR "impaired*" OR "impair*" OR "Cognitive Defect" OR "Cognitive Dysfunction" OR "Cognitive Dysfunction*" OR "Neurodevelopmental Disorders" OR "neurodevelopmental disorder" OR "Mental Health" OR "mental health" OR "Emotion" OR "Emotions" OR "Emotion*" OR "depression" OR "depress*" OR "anxiety" OR "anxiet*") OR SU("Developmental Disorder" OR "Developmental Disabilities" OR "Developmental disability" OR "Child Development Disorder" OR "Child Development Disorders" OR "Developmental Delay Disorders" OR "Developmental Delay Disorder" OR "Developmental Delay" OR "Child Development Deviations" OR "Child Development Deviation" OR "Autism" OR "Autism" OR "autistic" OR "asperger" OR "asperger*" OR "Attention Deficit Disorder" OR "ADDH" OR "ADHD" OR "Attention Deficit Disorder" OR "Attention Deficit Hyperactivity " OR "Hyperkinetic Syndrome" OR "Minimal Brain Dysfunction" OR "Learning Disorder" OR "Learning Disabilities" OR "Learning disability" OR "Dyscalculia" OR "Dyslexia" OR "Learning Disorder" OR "Learning Disorders" OR "Intellectual Impairment" OR "Disability" OR "Disabilities" OR "disab*" OR "special needs" OR "special need" OR "handicap" OR "handicaps" OR "handicapped" OR "handicap*" OR "impairment" OR "impairments" OR "impaired*" OR "impair*" OR "Cognitive Defect" OR "Cognitive Dysfunction" OR "Cognitive Dysfunction*" OR "Neurodevelopmental Disorders" OR "neurodevelopmental disorder" OR "Mental Health" OR "mental health" OR "Emotion" OR "Emotions" OR "Emotion*" OR "depression" OR "depress*" OR "anxiety" OR "anxiet*") OR MA("Developmental Disorder" OR "Developmental Disabilities" OR "Developmental disability" OR "Child Development Disorder" OR "Child Development Disorders" OR "Developmental Delay Disorders" OR "Developmental Delay Disorder" OR "Developmental Delay" OR "Child Development Deviations" OR "Child Development Deviation" OR "Autism" OR "Autism" OR "autistic" OR "asperger" OR "asperger*" OR "Attention Deficit Disorder" OR "ADDH" OR "ADHD" OR "Attention Deficit Disorder" OR "Attention Deficit Hyperactivity " OR "Hyperkinetic Syndrome" OR "Minimal Brain Dysfunction" OR "Learning Disorder" OR "Learning Disabilities" OR "Learning disability" OR "Dyscalculia" OR "Dyslexia" OR "Learning Disorder" OR "Learning Disorders" OR "Intellectual Impairment" OR "Disability" OR "Disabilities" OR "disab*" OR "special needs" OR "special need" OR "handicap" OR "handicaps" OR "handicapped" OR "handicap*" OR "impairment" OR "impairments" OR "impaired*" OR "impair*" OR "Cognitive Defect" OR "Cognitive Dysfunction" OR "Cognitive Dysfunction*" OR "Neurodevelopmental Disorders" OR "neurodevelopmental disorder" OR "Mental Health" OR "mental health" OR "Emotion" OR "Emotions" OR "Emotion*" OR "depression" OR "depress*" OR "anxiety" OR "anxiet*") OR AB("Developmental Disorder" OR "Developmental Disabilities" OR "Developmental disability" OR "Child Development Disorder" OR "Child Development Disorders" OR "Developmental Delay Disorders" OR "Developmental Delay Disorder" OR "Developmental Delay" OR "Child Development Deviations" OR "Child Development Deviation" OR "Autism" OR "Autism" OR "autistic" OR "asperger" OR "asperger*" OR "Attention Deficit Disorder" OR "ADDH" OR "ADHD" OR "Attention Deficit Disorder" OR "Attention Deficit Hyperactivity " OR "Hyperkinetic Syndrome" OR "Minimal Brain Dysfunction" OR "Learning Disorder" OR "Learning Disabilities" OR "Learning disability" OR "Dyscalculia" OR "Dyslexia" OR "Learning Disorder" OR "Learning Disorders" OR "Intellectual Impairment" OR "Disability" OR "Disabilities" OR "disab*" OR "special needs" OR "special need" OR "handicap" OR "handicaps" OR "handicapped" OR "handicap*" OR "impairment" OR "impairments" OR "impaired*" OR "impair*" OR "Cognitive Defect" OR "Cognitive Dysfunction" OR "Cognitive Dysfunction*" OR "Neurodevelopmental Disorders" OR "neurodevelopmental disorder" OR "Mental Health" OR "mental health" OR "Emotion" OR "Emotions" OR "Emotion*" OR "depression" OR "depress*" OR "anxiety" OR "anxiet*")) AND (TI("Caregiver" OR "Caregivers" OR "Caregiver" OR "caregiv*" OR "carer" OR "carers" OR "Parent" OR "Parents" OR "Parent" OR "parent*" OR "Family" OR "family" OR "families" OR "mother" OR "mothers" OR "mother*" OR "father" OR "fathers" OR "father*" OR "sibling" OR "sibling" OR "sibling*" OR "Sisters" OR "Sister" OR "Brothers" OR "Brother" OR "Home Care" OR "home care") OR SU("Caregiver" OR "Caregivers" OR "Caregiver" OR "caregiv*" OR "carer" OR "carers" OR "Parent" OR "Parents" OR "Parent" OR "parent*" OR "Family" OR "family" OR "families" OR "mother" OR "mothers" OR "mother*" OR "father" OR "fathers" OR "father*" OR "sibling" OR "sibling" OR "sibling*" OR "Sisters" OR "Sister" OR "Brothers" OR "Brother" OR "Home Care" OR "home care") OR MA("Caregiver" OR "Caregivers" OR "Caregiver" OR "caregiv*" OR "carer" OR "carers" OR "Parent" OR "Parents" OR "Parent" OR "parent*" OR "Family" OR "family" OR "families" OR "mother" OR "mothers" OR "mother*" OR "father" OR "fathers" OR "father*" OR "sibling" OR "sibling" OR "sibling*" OR "Sisters" OR "Sister" OR "Brothers" OR "Brother" OR "Home Care" OR "home care") OR AB("Caregiver" OR "Caregivers" OR "Caregiver" OR "caregiv*" OR "carer" OR "carers" OR "Parent" OR "Parents" OR "Parent" OR "parent*" OR "Family" OR "family" OR "families" OR "mother" OR "mothers" OR "mother*" OR "father" OR "fathers" OR "father*" OR "sibling" OR "sibling" OR "sibling*" OR "Sisters" OR "Sister" OR "Brothers" OR "Brother" OR "Home Care" OR "home care")) AND TX("Child" OR "child" OR "children" OR "Infant" OR "infant" OR "infants" OR "infancy" OR "newborn" OR "newborns" OR "new born" OR "new borns" OR "neonate" OR "neonates" OR "neonatal" OR "neo nate" OR "neo nates" OR "neo natal" OR "neonatology" OR "NICU" OR "premature" OR "prematures" OR "pre mature" OR "pre matures" OR "preterm" OR "pre term" OR "postnatal" OR "post natal" OR "baby" OR "babies" OR "suckling" OR "sucklings" OR "toddler" OR "toddlers" OR "childhood" OR "schoolchild" OR "schoolchildren" OR "childcare" OR "child care" OR "young" OR "youngster" OR "youngsters" OR "preschool" OR "pre school" OR "kid" OR "kids" OR "boy" OR "boys" OR "girl" OR "girls" OR "Adolescent" OR "adolescent" OR "adolescents" OR "adolescence" OR "pre adolescent" OR "pre adolescents" OR "pre adolescence" OR "schoolage" OR "schoolboy" OR "schoolboys" OR "schoolgirl" OR "schoolgirls" OR "pre puber" OR "pre pubers" OR "pre puberty" OR "prepuber" OR "prepubers" OR "prepuberty" OR "puber" OR "pubers" OR "puberty" OR "puberal" OR "teenager" OR "teenagers" OR "teens" OR "youth" OR "youths" OR "underaged" OR "under aged" OR "Pediatrics" OR "Pediatric" OR "Pediatrics" OR "Paediatric" OR "Paediatrics" OR "PICU" OR "child" OR children* OR schoolchild* OR "infant" OR "infants" OR "infancy" OR adolesc* OR pediat* OR paediat* OR neonat* OR toddler* OR "teen" OR "teens" OR teenager* OR preteen* OR newborn* OR postneonat* OR postnatal* OR "puberty" OR preschool* OR suckling* OR "juvenile" OR "new born" OR "new borns" OR new born* OR neo nat* OR neonat* OR perinat* OR underag* OR "under age" OR "under aged" OR youth* OR kinder* OR pubescen* OR prepubescen* OR "prepuberty" OR "school age" OR "schoolage" OR "school ages" OR schoolage* OR "one year old" OR "two year old" OR "three year old" OR "four year old" OR "five year old" OR "six year old" OR "seven year old" OR "eight year old" OR "nine year old" OR "ten year old" OR "eleven year old" OR "twelve year old" OR "thirteen year old" OR "fourteen year old" OR "fifteen year old" OR "sixteen year old" OR "seventeen year old" OR "eighteen year old" OR "1 year old" OR "2 year old" OR "3 year old" OR "4 year old" OR "5 year old" OR "6 year old" OR "7 year old" OR "8 year old" OR "9 year old" OR "10 year old" OR "11 year old" OR "12 year old" OR "13 year old" OR "14 year old" OR "15 year old" OR "16 year old" OR "17 year old" OR "18 year old" OR "two years old" OR "three years old" OR "four years old" OR "five years old" OR "six years old" OR "seven years old" OR "eight years old" OR "nine years old" OR "ten years old" OR "eleven years old" OR "twelve years old" OR "thirteen years old" OR "fourteen years old" OR "fifteen years old" OR "sixteen years old" OR "seventeen years old" OR "eighteen years old" OR "2 years old" OR "3 years old" OR "4 years old" OR "5 years old" OR "6 years old" OR "7 years old" OR "8 years old" OR "9 years old" OR "10 years old" OR "11 years old" OR "12 years old" OR "13 years old" OR "14 years old" OR "15 years old" OR "16 years old" OR "17 years old" OR "18 years old"))

### Ichushi

((((遠隔看護/TH or 遠隔看護/AL or テレナーシング/AL) or ((遠隔医療/TH or 遠隔/TA or インターネット/TH or 障害者用コミュニケーションエイド/TH or インターネット/TA or オンライン/TA or ICT/TA or ウェブ/TA) and (医療/TA or 診療/TA or 看護/TA or SB=看護 or 看護介入/TH or 看護職/TH or 家族看護/TH or 保健医療サービス提供/TH or 在宅介護/TH or 在宅介護支援サービス/TH or 在宅ケア支援/AL))) and (神経発達症/TH or 神経発達症/AL or 発達障害/TA or 学習障害/TA or 自閉症/AL or 自閉スペクトラム/AL or アスペルガー/AL or ADHD/AL or 注意欠如/AL or 多動/AL or 知的障害/TH or 知的障害/AL or 医療的ケア/TH or 医療的ケア/TA or 障害者/TH or 重症心身障害者/TH or 重症心身障害/TA) and ((小児/TH or 青年/TH or 学生/TH or 障害児/TH or 子ども/TA or 小児/TA or 乳児/TA or 幼児/TA or 児童/TA or 就学/TA or 思春期/TA or 生徒/TA or 学生/TA or 青年/TA or 障害児/TA) or (介護者/TH or 両親/TH or 家族/TH or 家族/TA or 父親/TA or 母親/TA or 親子/TA or きょうだい/TA or 兄弟姉妹/TA)))) and (DT=1900:2022 and PT=原著論文)

note: In order to make the concept clearer and more comprehensive, we have changed some of the search strategies from the original plan.
